# Inspiration or risk? How social media marketing of plant-based meat affects young people’s purchase intention

**DOI:** 10.3389/fpsyg.2022.971107

**Published:** 2022-10-10

**Authors:** Tingting Li, Desheng Wang, Zhihao Yang

**Affiliations:** Department of Marketing, School of Management, Shandong University, Jinan, China

**Keywords:** plant-based meat, social media marketing, dual-system theory, cognitive fluency, customer inspiration, perceived risk, brand community identity

## Abstract

As an alternative protein product to animal meat, plant-based meat is considered to play an essential role in improving animal welfare and protecting the environment. However, why do a few consumers choose plant-based meat but others do not? Despite the increasing research on plant-based meat marketing, little is known about the psychological mechanism by which plant-based meat marketing affects consumers’ purchasing decisions. We utilize dual-system theory to understand how social media marketing of plant-based meat influences cognitive fluency, customer inspiration, perceived risk, and purchase intention. Four studies (i.e., Studies 1, 2, 3, and 4) show that social media marketing can increase young people’s purchase intention of plant-based meat more than traditional marketing. In Studies 1 and 2, increased intensity of social media marketing can enhance young people’s cognitive fluency and further promote purchase intention. Study 3 explores how cognitive fluency relates to purchase intention through two psychological mechanisms. We suggest that a higher level of cognitive fluency increases customer inspiration and improves purchase intention. However, a lower level of cognitive fluency reduces purchase intention by increasing perceived risk. Study 4 manipulated members’ in-group or out-group status to show a boundary condition for the effect of brand community identity on purchase intention. These studies provide insight into how brand marketers can use social media to promote consumer inspiration and advertising engagement, how managers can offer fluency-increasing mechanisms to ensure a low level of perceived risk, and how enterprise practitioners may want to consider brand community publicity to attract out-group members.

## Introduction

Food production and consumption represent one of the most significant contributors to environmental issues (Vermeulen et al., 2012). Recent years have seen increasing interest in research on plant-based food marketing because it is vital for environmental protection and improvement in animal welfare. For example, using soy protein concentrate (SPC) as raw material, plant-based meat now provides prospects for improving human health, and reducing environmental pollution and meat consumption (Grebenyuk and Ravin, 2017). China has been identified as a prime country to conduct consumer surveys on plant-based meat since it has the highest population and the rising economy in the world (Bryant and Julie, 2018). Meanwhile, with increasing demand for meat consumption coming from developing countries (Bryant et al., 2018), research on consumer acceptance of plant-based meat could help China achieve carbon neutrality.

Academicians have paid attention to consumer attitudes and behaviors toward plant-based meat (Yu et al., 2014; Bryant and Sanctorum, 2021). McCarthy et al. (2015) verified that Chinese purchase intention of plant-based meat was driven by altruistic concerns (i.e., environment and animal welfare concerns) and self-interest (i.e., health concerns and food safety concerns). Shan et al. (2022) also investigated that the Chinese were more willing to buy artificial meat under positive information. Other studies showed that the growth of plant-based meat sales was significant in the last few years, and younger had relatively stronger preferences for plant-based meat than farm-raised meat (Loo et al., 2020; Zhao et al., 2022). Although previous studies have provided essential evidence on the factors of plant-based meat’ consumption, there is a lack of exploration in specific contexts, mainly in social media marketing (Qutteinq et al., 2019; He et al., 2020). Social media marketing is more in line with the habits of young people, significantly improving the interaction between consumers and plant-based meat (Moreira et al., 2021). Specifically, consumers rely on expert opinions, friend recommendations, and advertising on social media to acquire knowledge of plant-based meat and be influenced by them (Thompson et al., 2016). Therefore, research is needed to explore these views in more detail and understand whether and how social media marketing may influence young people’s cognitions and attitudes toward plant-based meat.

The rise of social media has blurred the boundary between brand operation and media management, enabling timely dissemination, sharing, and user interaction of product information (Hanna et al., 2011; Aliosha et al., 2013; Appel et al., 2019). Baetzgen and Tropp (2013) pointed out that product communication is not limited to traditional advertising, brand implantation, soft text, sponsorship, and content marketing but has also been widely used in social media. In this way, enterprises push plant-based meat to social media through diversified advertising methods, expecting to bring a low-cost and significant flow interaction effect, thus expanding brand influence and value (Yu et al., 2017). In addition, based on high participation and interaction of social media, enterprises can receive consumers’ feedback and suggestions in real-time and understand consumers’ attitudes toward plant-based meat as consumers are no longer merely passive recipients in the marketing exchange process (Felix et al., 2016). Despite what academics and practitioners have studied on the advantages of social media marketing, the psychological mechanisms consumers interact with plant-based meat are unclear (Appel et al., 2019). In this research, we examine whether social media marketing of plant-based meat motivates young people to interact more and impacts their cognition, emotion, and purchase intention. Furthermore, we inspect how cognitive fluency influences young people’s engagement with plant-based meat marketing. The resulting emotional and cognitive states have the potential to provide valuable information on plant-based meat social media advertising success.

Interestingly, research findings also suggest that consumers may experience differential positive and negative cognitions from plant-based meat (Yu et al., 2014; McCarthy et al., 2015). In general, plant-based meat is considered an increasingly important tool in reducing the consumption of animal products for environmental, public health, and ethical reasons, affecting consumers’ positive cognitions and attitudes (McCarthy et al., 2015). Conversely, Bryant et al. (2018) indicated that consumers held more risks because they had less experience with plant-based meat. Perceived risk is easy to diminish consumers’ purchase intention and weaken other factors’ positive impact on consumer behavior (Ueland et al., 2012). In addition, plant-based meat has a low repurchase rate while being sought after by the middle and young, suggesting that there have been many instances wherein consumers have raised social and health concerns about such products (Rojas-Méndez et al., 2012). The individuals have opposite perceptions of inspiration and risk of plant-based meat, further affecting their purchase intention (Radford and Bloch, 2011). Despite the above conclusions, none of the studies have measured plant-based meat consumption with an integrated mechanism of cognition in the social media marketing context. Therefore, understanding how can enhance acceptance of plant-based meat through different psychological processes is required.

Thus, our research seeks to answer the following questions to advance theory and research on plant-based meat marketing: (1) Whether marketing through social media can promote young people’s purchase intention? Moreover, (2) If so, by what mechanism is it achieved? We draw on dual-system processing theory to answer those questions, which states that people have two independent information processing systems, including sensibility (quick, intuitive, and effortless) and rationality (slow, analytical, and deliberate; Alter et al., 2007). First, this theory provides a general framework for understanding young people’s cognitive processes and decision-making when evaluating plant-based products (Study 1 and 2: social media marketing intensity, cognitive fluency, and purchase intention). Furthermore, dual-system processing theory suggests that two mechanisms (customer inspiration and perceived risk) account for the effects of social media marketing on purchase intention (Study 3: customer inspiration and perceived risk as mediating mechanisms). We also go one step further and examine a vital boundary condition of whether brand community identity influences young people’s purchasing decisions on plant-based meat (Study 4: the moderating role of in-group and out-group). We do not pre-register hypotheses and will conduct exploratory studies to reason and test the above questions. The resulting model allows us to answer why, how, and for whom social media marketing of plant-based meat is effective.

Overall, our paper offers three key contributions. First, we explore the relationship between social media marketing of plant-based meat and young people’s purchase intention. This advances research on plant-based meat marketing by identifying a new psychological mechanism that clarifies the cognitive and decision-making processes of young people. Second, and perhaps more importantly, we show that customer inspiration and perceived risk are the two paths of consumer perception. This challenges the assumption that marketing of plant-based meat and purchase intention will constantly interact positively or negatively (Yu et al., 2014; McCarthy et al., 2015) and also enriches the classic dual-system processing theory. Third, we demonstrate that consumers’ identification with the brand community can influence whether customers are inspired or perceived as at risk. Our work enriches the range of mechanisms associated with plant-based meat research, answering calls to examine the boundary condition of social media marketing of plant-based products.

## Literature review and hypotheses

### Social media marketing of plant-based meat and young people’s purchase intention

Social media marketing refers to using social media, such as blogs, MicroBlog, WeChat, and shared forums, to enhance the visibility and recognition of enterprises, brands, and products (Krishen et al., 2016). Celebrities have linked plant-based meat to fitness and shared their reviews on social media, making this new product gradually known to Chinese consumers. Appel et al. (2019) showed that most consumers could benefit from product reviews and sharing on social media. With less knowledge about plant-based meat, young people are not aware of its utilitarian and hedonic functions when they first come into contact with plant-based meat. However, social media marketing displays plant-based meat’ features and attributes through text descriptions, data indicators, pictures, animation, celebrities’ live broadcasts, and user evaluations, allowing individuals to learn more about plant-based meat (Couldry and Markham, 2007). Meanwhile, social media allows young people to interact with friends and browse product information posted by celebrities. Young people prefer to purchase products based on peers’ or celebrities’ opinions rather than ads shared by a brand (Yu et al., 2017; Murphy et al., 2020). Therefore, we suggest that compared with traditional advertising, social media marketing enables young people to be aware of the environmental and health concerns of plant-based meat, and timely sharing with peers also enhances social attributes, thus increasing purchase intention.

Studies also showed that unfamiliarity gave the Chinese lower acceptance rates of plant-based meat (Bryant et al., 2018). Meanwhile, curiosity was regarded as one of the strongest motivating factors for purchasing plant-based meat (Hwang et al., 2020). This paper predicts that with the increase in social media marketing intensity, young people are more willing to buy plant-based meat. On the one hand, intensive marketing means young people have more opportunities to learn product details, reducing unfamiliarity with plant-based meat. On the other hand, social media allows young people to connect with friends extensively, presenting pictures or sharing experiments with plant-based meat (Davis, 2012; Holmberg, 2016). Young people’s curiosity will be piqued when peers talk more about plant-based meat (Yau and Reich, 2018; Hwang et al., 2020). Based on this, we predict the following:

*Hypothesis 1*: Social media marketing significantly enhances young people’s purchase intention toward plant-based meat compared with traditional advertising. In addition, there is a positive relationship between social media marketing intensity and purchase intention of plant-based meat.

### Dual-system processing theory

We draw upon dual-system processing theory to further explain young people’s cognitive process to plant-based meat. Cognitive information processing (CIP) theory focuses on how individuals process and interpret information in social situations (Crick and Dodge, 1994), which is the basis of dual-system processing theory. Lemerise and Dodge (2000) developed the CIP theory to demonstrate how individuals encode information cues, classify goals, extract responses, make decisions, and implement behaviors. Dual-system processing theory is the extension of CIP theory. Different from the CIP theory, which comprehensively summarizes cognitive and decision-making processes, the dual-system processing theory focuses on explaining the other decision-making mechanisms of consumers (Alter et al., 2007). The basic tenet is that individuals have both perceptual (system 1) and rational (system 2) decision-making systems and decide which procedure plays a dominant role according to the specific context. According to dual-system processing theory, one of the conditions for system 1 or system 2 to be activated is the individual’s experience of information processing difficulty (Alter et al., 2007). If consumers perceive information processing to be easy, system 1 is more likely to be activated, leading to intuitive, effortless, and rapid processing (Alter et al., 2013). Conversely, if consumers perceive information processing to be complex, system 2 is more likely to be activated, and individuals put more mental effort into it and turn to analytical thinking (Shen and Rao, 2016).

According to the dual-system processing theory, this paper believes that young people first form different product cognition of plant-based meat. The fluency of information processing determines which system is activated faster, and further stimulates customer inspiration and perceived risk. Therefore, we identify three main factors to explain the acceptance process of plant-based meat: (1) cognitive fluency, (2) customer inspiration, and (3) perceived risk.

### The mediating effect of cognitive fluency

Cognitive fluency reflects an individual’s subjective experience about information processing, mainly referring to low-level processing (Lee and Labroo, 2004; Shen and Rao, 2016). Information processing produces cognitive and emotional consumption, so consumers are unwilling to spend extra energy on cognition (Aydinli et al., 2014). Hu et al. (2017) demonstrated this opinion by suggesting that product familiarity reduces cognitive load. Since plant-based meat advertisements usually contain non-empirical information, processing may increase consumer cognitive load and lead to adverse communication effects. However, compared with traditional advertising, social media marketing enables youngers to interact with plant-based meat promptly and form specific cognition (Liu et al., 2021). The resulting familiarity increases cognitive fluency. In addition, Nunes et al. (2015) also showed that repeated promotions, easy-to-understand instructions, product appearance, and consumer visual habits induced cognitive fluency. That is to say, the increase in marketing intensity can improve young people’s cognitive fluency.

Cognitive fluency will further influence young people’s judgment of product authenticity, thus affecting their decision-making. When cognitive fluency is high, young people do not need to consume many cognitive resources to process information; otherwise, they need to invest more cognitive efforts (Thompson and Ince, 2013; Graf et al., 2018). For example, Labroo and Pocheptsova (2016) showed that consumers increased their liking for products due to the fluent processing of product descriptions, designs, and advertisements. However, when the cognition is not fluent, consumers are less favorable of the product (Jiang et al., 2016). In the context of social media marketing, we predict that cognitive fluency positively affects young people’s purchase intention, for the following reasons: (1) Fluency experience motivates consumers to maintain more knowledge about plant-based meat, while familiarity enhances consumers’ purchase intention (Graf et al., 2018); (2) The smoother the advertising message, the more easily consumers are persuaded (Seo and Scammon, 2017). Social media provides fewer information gaps, increasing advertisements’ persuasiveness (Kwan et al., 2017). On this basis, we hypothesize:

*Hypothesis 2*: Cognitive fluency is mediating between the marketing intensity of social media and young people’s purchase intention for plant-based meat.

### Customer inspiration and perceived risk as mediating mechanisms

Social media marketing affects purchase intention by changing young people’s cognitive fluency with plant-based meat. However, cognitive fluency is a leading factor in consumers’ purchasing decisions but not the ultimate determinant (Parker et al., 2016). Drawing from dual-system processing theory, consumers engage in deep and abstract information processing when cognitive fluency is low. Individuals increase emotional pleasure and imagination when cognitive fluency is high (Carr et al., 2016; Labroo and Pocheptsova, 2016). Thus, we suggest that the subsequent impact of cognitive fluency on purchase intention depends on whether customer inspiration or perceived risk is dominant.

Classical inspiration theory holds that new ideas from outside stimulate the generation of customers’ inspiration and put individuals in an incentive state to put their ideas into practice (Thrash and Elliot, 2004). Böttger et al. (2017) further proposed that customer inspiration includes “inspired-by” and “inspired-to” two stages. According to the dual-system processing theory, smooth information triggers system 1 so consumers conduct intuitive heuristic reasoning mode and respond quickly (Alter et al., 2013). Customer inspiration represents this temporary motivational state of consumers. Although system 1 and system 2 interact, smooth information will trigger system 1 and activate customer inspiration more. Next, customer inspiration can effectively predict consumers’ attitudes and behavior (Böttger et al., 2017). Böttger et al. (2017), for example, designed a series of experiments in which three subjects were asked to shop online with product descriptions of different inspirations. The research found that products with high inspiration led to more purchase intentions.

Social media advertisements provide young people with detailed and rich information about plant-based meat, making them have an intuitive feeling about plant-based meat (Liang et al., 2016; Ho et al., 2017). This paper predicts that the greater the fluency of young people’s perception, the easier it is to promote their immediate imagination on plant-based meat’s taste and environmental protection function (Chen and Zheng, 2015). In addition, smooth information is often accompanied by positive emotions, further promoting the inspiration of young people, such as detached experience, joy shared with friends, and surprise, triggering unplanned purchase intentions (Fu and Elliott, 2013; Wang et al., 2014). Once young people are inspired, their subsequent consumption behavior becomes more spontaneous and impulsive. On this basis, we hypothesize cognitive fluency’s positive, indirect effect on purchase intention through customer inspiration.

*Hypothesis 3*: Compared with a lower level of cognitive fluency, a higher level of cognitive fluency is more likely to generate customer inspiration and improve purchase intention.

Like customer inspiration, the perceived risk exists in the evaluation and decision-making process, thus becoming an important tool to reveal individual behavior and decision-making rules (Garretson and Clow, 1999; Mitchell, 1999). It is a multidimensional concept, including financial risk, performance risk, physical risk, social risk, psychological risk, and time risk (Stone and Grønhaug, 1993). However, perceived risk may provide contrary theoretical support for our hypothesis. According to the dual-system processing theory, disfluent information triggers system 2, at which point consumers tend to conduct deeper, more abstract, and more careful analysis (Shen and Rao, 2016). This will significantly increase individuals’ risk perception, affecting purchase intention (Hirunyawipada and Paswan, 2006). Empirical studies have also proved this point. For example, Ueland et al. (2012) indicated in the BRA model that perceived risk can easily reduce consumers’ purchase intention and weaken the positive impact of other factors on consumer behavior.

In this study, plant-based meat uses new technology or formula but is expensive, which will increase financial risk for young people (Dholakia, 2001; Bryant and Julie, 2018). Chinese have a diet of traditional animal meat, and the composition of plant-based meat may cause young people’s perception of health risks. A variety of social media information results in cognitive load, increasing young people’s perception of health risks (Hirunyawipada and Paswan, 2006). Beyond that, consumers need more emotion and cognition to sort out product information and suffer from uncertain results, which will increase psychological risk. However, consumers would actively avoid information with poor cognitive fluency (Seo and Scammon, 2017). When the risks of plant-based meat cause too much anxiety, young people choose not to purchase them. In summary, we propose the hypothesis that when cognitive fluency is low, young people will generate more perceived risks while arousing inspiration, thus reducing the purchase intention of plant-based meat.

*Hypothesis 4*: Compared with a higher level of cognitive fluency, a lower level of cognitive fluency is more likely to stimulate perceived risk, thus reducing purchase intention.

### The moderating effect of brand community identity

While social media enhances users’ willingness to share, it also makes users’ behavior more susceptible to influence (Yau and Reich, 2018). Consumers, especially the young, also refer to and follow their peers’ posts. Thus, this paper uses brand community identity to describe consumer status. Brand community is a social relationship formed by brand lovers without geographical restrictions. Community members usually have a solid psychological identity, connections, or belonging to a brand community (Chadborn and Reysen, 2018). Notably, most members are existing or potential users of the brand, who constitute the core users of the brand (Thompson et al., 2016). Based on this, we use in-group and out-group to further distinguish consumers’ situation.

In examining the effect of brand identity on community members, previous research found that identity strength affected the motivation individuals received in groups (Kleine et al., 1993). Social and sharing motivations guide in-group members’ purchase intention, significantly increasing inspiration’s intensity, frequency, and emotional component (Algesheimer et al., 2005). In addition, in-group members perceive themselves as part of the brand community. The dependence generated by familiarity with products makes them insensitive to plant-based meat’ risks. Indeed, they are more likely to imagine themselves sharing product-related posts or shopping experiences with friends in a positive mood (Chadborn and Reysen, 2018). For these reasons, we expect that social media marketing will lead to smoother cognition of plant-based meat among in-group members, generating more customer inspiration and thus increasing purchase intention. Unlike in-group members who tend to imagine, out-group consumers are more cautious and monitor their environment continuously to avoid mistakes (Kardes et al., 2004). They have a common understanding of the functions and attributes of plant-based meat and do not have close relationships with community members. The lack of purchasing habits for such plant-based meat makes members fail to improve cognitive fluency and customer inspiration. In addition, unfamiliar information also increases out-group consumers’ perceived risk. Accordingly:

*Hypothesis 5*: In the social media marketing condition, when consumers are in-group, they are more likely to generate (a) higher cognitive fluency and (b) customer inspiration, further enhancing purchase intention.

*Hypothesis 6*: When consumers are out-group, they are more likely to generate (a) lower cognitive fluency and (b) perceived risk, further reducing purchase intention.

**Figure 1:**
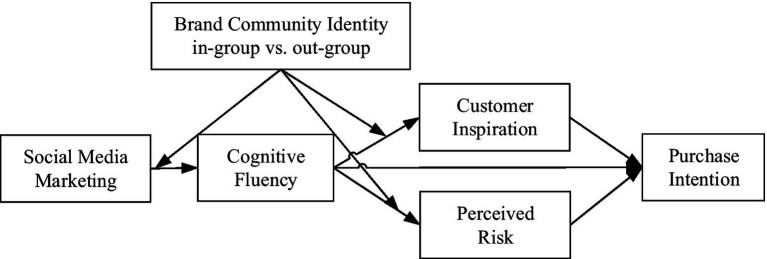
The conceptual framework.

In summary, we presented our conceptual framework in Figure 1.

## Study design and result analysis

### Study overview

We conducted four studies to examine how social media marketing of plant-based meat affects young people’s purchase intention and the underlying mechanism by testing the mediating roles of cognitive fluency, customer inspiration, and perceived risk. In Study 1, we first used authentic plant-based meat brands to provide preliminary tests of our theorizing, demonstrating that plant-based meat’s social media marketing (vs. traditional marketing) had a greater impact on purchase intention. In Study 2, we used an authentic plant-based meat brand (Plant Diary) in China as a stimulus to establish the primary main effect of social media marketing on purchase intention and the mediation effect of cognitive fluency through a lab study. Study 3 further tested the mediating roles of customer inspiration and perceived risk. Participants formed, through direct experience, an authentic individual preference for a product. Study 4 tested the moderating effect of brand community identity. These four studies tested our theoretical framework’s psychological mechanism and boundary condition. We designed these four studies by referring to the experimental methods and sample sizes in previous studies, and reported the stimulus materials, manipulation, data exclusion, and methods. This paper involved several manipulative experiments, requiring complex design and offline labs. Therefore, we mainly adopted small sample study, which would also be explained as a limitation.

### Study 1: Main effect

#### Design and method

Study 1 aimed to test whether social media marketing can increase young people’s purchase intention of plant-based meat more than traditional marketing. Firstly, we collected ten emergings, authentic plant-based meat brands (such as Plant Diary, Beyond Meat, Protein Meat, Harvest Gourmet, Future Meat, and Qishan Food) from China’s most extensive shopping site Taobao.com. We then invited ten experts and Ph.D. students in marketing to discuss the ten brands we collected. The stimulus materials should meet two conditions: brand familiarity was low, and product types included in the brand were widely accepted by customers. After discussion, we chose Plant Diary, Beyond Meat, and Protein Meat as the experimental stimulus and designed advertising content for each brand. Table 1 shows the details of the stimulus materials and the manipulation methods of traditional and social media marketing. We simulated the interaction process of consumers in the context of social media marketing with “participants can make comments after posts.” Other experimental conditions were completely the same except for advertising methods. In addition, we would initially test consumers’ brand preference degree to exclude the influence of consumer preferences.

**Table 1:** Manipulation materials (Study 1).

Plant-based meat brand	Traditional marketing	Social media marketing
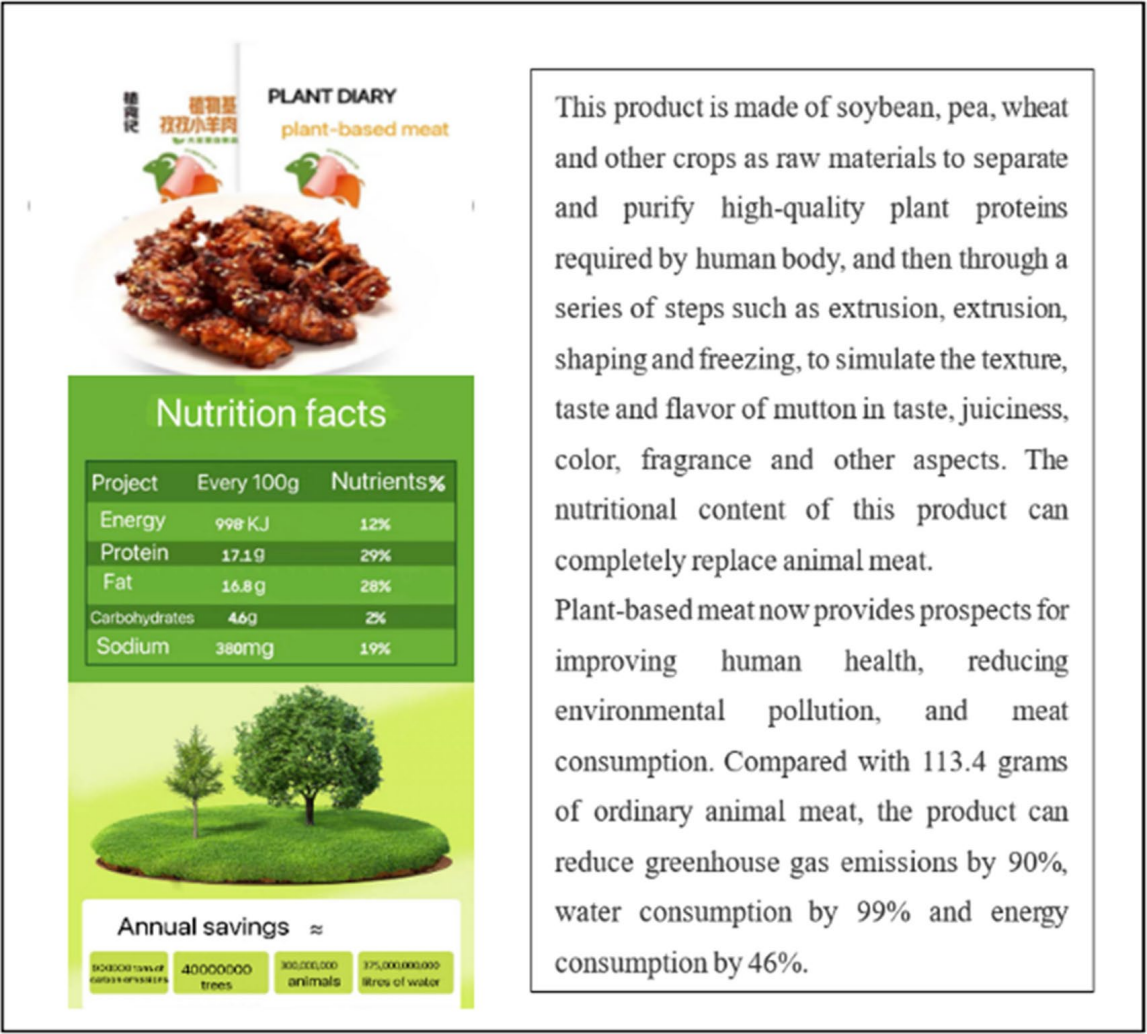	We provided the participants with a product poster via email about Plant Diary, introducing nutrition composition, taste, environmental protection concepts, and other contents of plant-based meat in the form of pictures and texts.	We provided the participants with a product promotion post about Plant Diary through WeChat, introducing nutrition composition, taste, environmental protection concepts, and other contents of plant-based meat in the form of pictures and texts. Participants can post comments after the post.
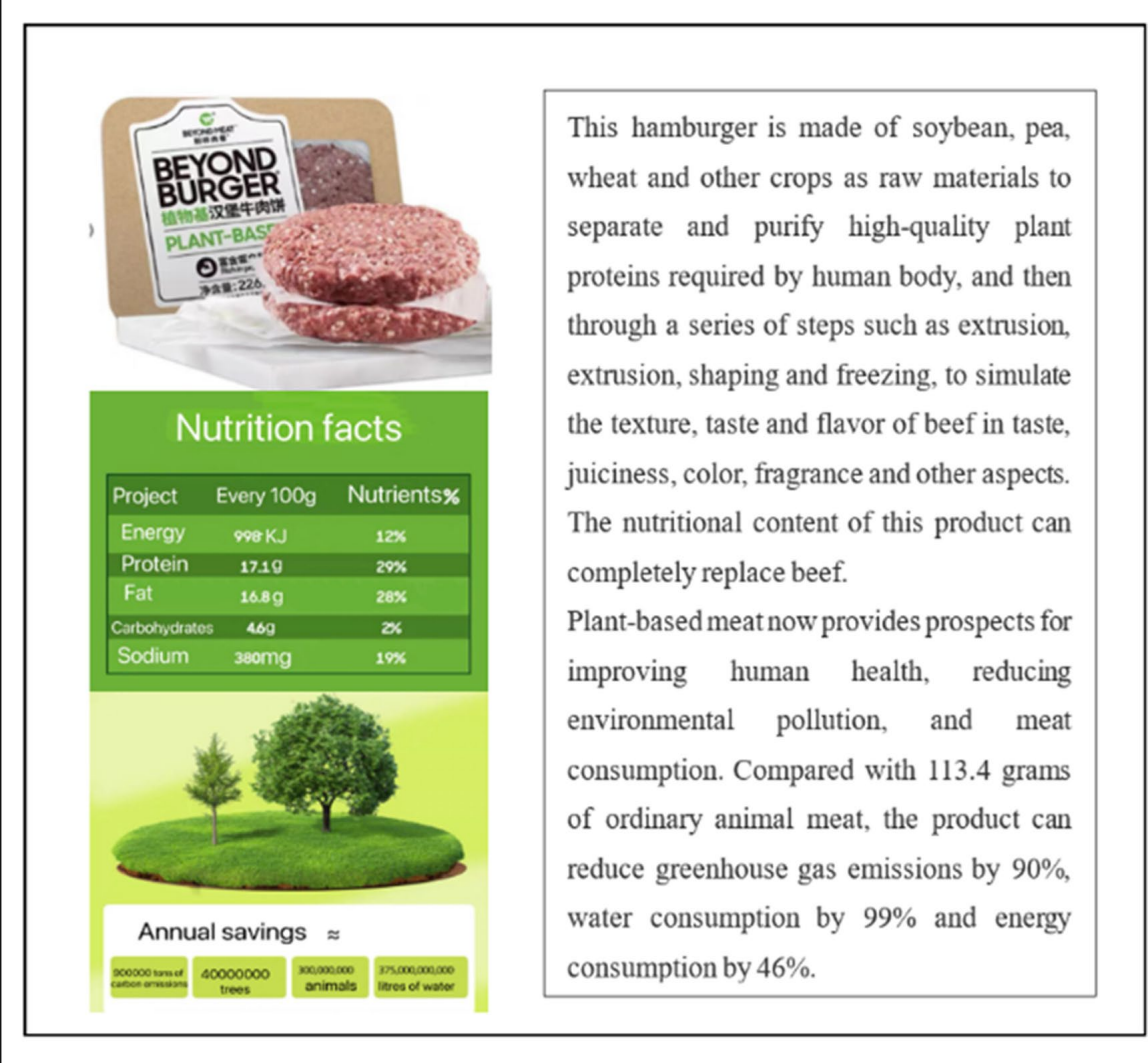	We provided the participants with a product poster via email about Beyond Meat, introducing nutrition composition, taste, environmental protection concepts, and other contents of plant-based meat in the form of pictures and texts.	We provided the participants with a product promotion post about Beyond Meat through WeChat, introducing nutrition composition, taste, environmental protection concepts, and other contents of plant-based meat in the form of pictures and texts. Participants can post comments after the post.
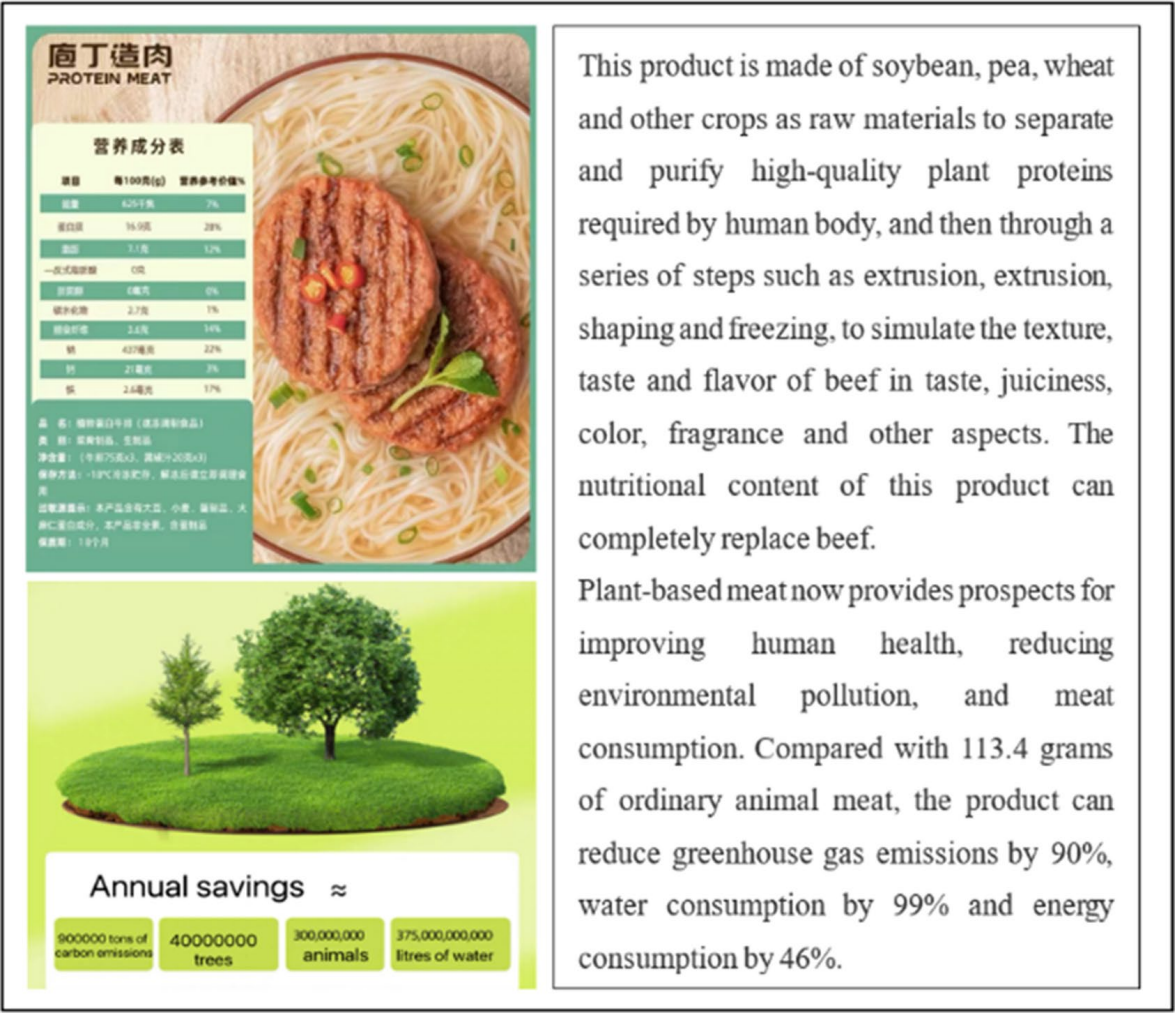	We provided the participants with a product poster via email about Protein Meat, introducing nutrition composition, taste, environmental protection concepts, and other contents of plant-based meat in the form of pictures and texts.	We provided the participants with a product promotion post about Protein Meat through WeChat, introducing nutrition composition, taste, environmental protection concepts, and other contents of plant-based meat in the form of pictures and texts. Participants can post comments after the post.

Data were collected for `4 days in May 2022 with a support of Questionnaire site, an online survey institution. We recruited 180 participants and evenly divided them into six groups (115 females, M_age_ = 26.50, ranging from 18 to 32 years old) for the test. We first tested participants for brand familiarity (“I’m familiar with the brand”; 1 = strongly disagree, 7 = strongly agree) and brand preference (“I love this brand”; 1 = strongly disagree, 7 = strongly agree). Then, we manipulated the three plant-based meat brands into six groups according to the manipulation method in Table 1. The participants were asked to read the material and answer the following question, “What kind of marketing do you think this is” (1 = traditional marketing, 7 = social media marketing). We also asked participants to rate the degree to which they agree with the following statement (Kim and Chung, 2013): “I am willing to buy this product” and “This product is what I want to buy” (1 = strongly disagree, 7 = strongly agree; α = 0.93).

#### Results and discussion

##### Manipulation check

T-test results showed significant differences between traditional marketing and social media marketing of Plant Diary (M_traditional_ = 2.20, M_social media_ = 5.70, *t* = −12.38, *p* < 0.001), Beyond Meat (M_traditional_ = 2.17, M_social media_ = 6.10, *t* = −15.81, *p* < 0.001), and Protein Meat (M_traditional_ = 2.01, M_social media_ = 5.87, *t* = −16.75, *p* < 0.001), indicating successful variable manipulation. Especially, there was no significant difference in brand familiarity and brand preference degree among each group. This suggested that the above three brands can be manipulated.

##### Purchase intention

The results showed a significant difference in purchase intention scores between the two groups. Social media marketing of Plant Diary (M = 4.90, SD = 1.56) was significantly higher than traditional marketing of Plant Diary (M = 3.47, SD = 1.53; *t* = 3.78, *p* < 0.001). The same was true for social media marketing of Beyond Meat (M = 5.17, SD = 1.29) and traditional marketing of Beyond Meat (M = 3.00, SD = 0.95; *t* = 6.89, *p* < 0.001) and social media marketing of Protein Meat (M = 5.00, SD = 1.14) and traditional marketing of Protein Meat (M = 3.17, SD = 1.29; *t* = 5.35, *p* < 0.001). By manipulating different advertising methods for three brands, we proved that social media marketing of plant-based meat could promote young people’s purchase intention more than traditional marketing.

### Study 2: Main effect and mediation effect

#### Design and method

Study 2 aimed to test whether cognitive fluency mediates the relationship between social media marketing intensity and purchase intention. We conducted a lab study to test these hypotheses. We invited students to experiment in a comprehensive university located in northern China, and finally recruited a separate sample consisting of 200 students and MBA (97 females, M_age_ = 25.40, ranging from 19 to 29 years old) in exchange for course credit.

Next, we chose Plant Diary as the stimulus and carried out this experiment in the lab. To test whether increased social media marketing intensity can improve consumers’ cognitive fluency and purchase intention toward plant-based meat, we designed the following experiment scenario: “Plant-based meat has become a popular choice in recent years. Using soy protein concentrate (SPC) as raw material, plant-based meat has become a substitute for traditional animal meat. Now there is a new brand of plant-based meat on the market.” Then, we provided the participants with a product post about Plant Diary through WeChat, introducing nutrition composition, taste, environmental protection concepts, and other contents in the form of pictures and texts. Participants were asked to post comments and read others’ posts on their phones. Especially, experimental stimulus, situation, process, and rewards for participants in study 2 were the same as in study 1.

Finally, participants were asked to fill in the questionnaire for marketing intensity (“you feel this company has a lot of marketing intensity”; 1 = strongly disagree, 7 = strongly agree), cognitive fluency through a four-item scale used by Dragojevic et al. (2017) (e.g., “I can easily read the information of the advertisement”; 1 = strongly disagree, 7 = strongly agree; α = 0.97), and purchase intention (Cronbach’s α = 0.91). Meanwhile, our study used product knowledge and brand preference as control variables to exclude their interference. Demographic information was also required. However, we did not include them in our analysis because of fewer differences among all samples. After excluding invalid questionnaires, there were 188 valid questionnaires (93 females, M_age_ = 25.17, SD = 3.02). Table 2 shows the demographic information of the samples.

**Table 2:** Demographic composition of the participants (Study 2).

Variables	Items	Number	Percentage
Sex	Male	95	51%
	Female	93	49%
Age	15–19 years old	5	3%
	20–24 years old	83	44%
	25–29 years old	100	53%
Education level	Undergraduate	66	35%
	Graduate student or more	122	65%

#### Results and discussion

First, ANOVA on purchase intention revealed a significant main effect of social media marketing intensity [*F*(1,187) = 376.25, *p* < 0.001]. Second, cognitive fluency positively affected consumers’ purchase intention [*F*(1,187) = 981.97, *p* < 0.001].

##### Mediation effect

We applied mediation analysis to test the expected underlying roles of cognitive fluency (Hayes, 2013; Model 4: 5000 bootstrapped samples). The results showed that the confidence interval of indirect effects (95% CI: 0.23–0.56, *p* < 0.01) did not include 0, which means the mediating effect of cognitive fluency was significant, and the effect score was 0.39. After controlling the mediating variables, marketing intensity also directly affected purchase intention (β = 0.40, Se = 0.14; 95% CI: 0.13–0.67, *p* < 0.01). The result is shown in Table 3. The indirect effect proved that cognitive fluency partly mediated the relationship between the intensity of social media marketing and young people’s purchase intention, supporting H1 and H2.

**Table 3:** Mediation effect of cognitive fluency (Study 2).

	Effect	SE	*t*	*p*	LLCI	ULCI
Direct effect	0.40	0.14	1.37	0.00	0.13	0.67
Indirect effect	0.39	0.12	-	-	0.23	0.56

### Study 3: Mediation effect of customer inspiration and perceived risk

#### Design and method

Study 3 aimed to test whether customer inspiration and perceived risk play a mediating role (H3 and H4). We adopted a one-factor, three-level (marketing intensity: once a week vs. three times a week vs. seven times a week), between-subjects design. These three levels of marketing intensity represented the low, medium, and high frequency, respectively. We also invited a blogger on WeChat to assist us in conducting this online experiment. This blogger had a community of more than 2000 members, mainly composed of university students, bank employees, college teachers, and company employees. Then, we sent research invitations to the members, and finally, 300 members were recruited to participate in study 3 (N = 300, 176 female, M_age_ = 26.90, ranging from 18 to 34 years). At this point, participants were randomly assigned to three WeChat groups, with an average of 100 each.

Study 3 selected Plant Diary from study 1 as a stimulus, and the intensity of social media marketing was manipulated. The blogger sent product information to three WeChat groups weekly, three times a week, and seven times a week, respectively. In particular, the conditions were the same except for the frequency at which sent the manipulated material ads. We initially measured the “brand familiarity” of the three groups and found that there was no significant difference (M_once_ = 1.30, M_three-times_ = 1.45, M_seven-times_ = 1.62, *p* > 0.05).

After a week, we asked participants to fill out a 7-point Likert scale for marketing intensity (“you feel this company has a lot of marketing intensity”; 1 = strongly disagree, 7 = strongly agree), cognitive fluency (Cronbach’s α = 0.89), and purchase intention (Cronbach’s α = 0.93). As for the measurement of customer inspiration, since “purchase intention” contains the content of “inspired-to,” we finally selected a five-item scale to focus on measuring the “inspired-by” of participants (e.g., “This product captured my imagination” and “This product has broadened my horizons”; Cronbach’s α = 0.91) (Thrash and Elliot, 2004; Böttger et al., 2017; Jian et al., 2021). We measured perceived risk through a five-item scale used by Stone and Grønhaug (1993) (e.g., “It may take me a lot of time to learn how to use this product” and “I do not think it makes economic sense to buy this product”; Cronbach’s α = 0.94; 1 = strongly disagree, 7 = strongly agree). The specific content of the scale is shown in Table 4. Meanwhile, study 2 required all participants to provide demographic information. Table 5 shows the demographic data of the samples. Participants received a small payment after completing the questionnaire. Finally, there were 288 valid questionnaires (173 females, M_age_ = 27.25, SD = 3.88).

**Table 4:** Contents of the scale.

Variable name	Item content
Cognitive fluency (Dragojevic et al., 2017)	I can easily read the information of the advertisement
	I can master the product knowledge conveyed in the advertisement
	I can easily understand the product information in the advertisement
	I can clearly grasp new product features
Customer inspiration (Thrash and Elliot, 2004; Böttger et al., 2017; Jian et al., 2021)	This product captured my imagination
	This product gave me a sudden new idea
	This product has broadened my horizons
	It made me discover something new
	The inspiration for this product is exciting
Perceived risk (Stone and Grønhaug, 1993)	It may take me a lot of time to learn how to use this product
	I do not think it makes economic sense to buy this product
	This product may cause me physical discomfort
	I’m worried that this product is not an effective solution to the problems I’m facing
	This product may cause me psychological discomfort
Purchase intention (Kim and Chung, 2013)	I am willing to buy this product
	This product is what I want to buy

**Table 5:** Demographic composition of the participants (Study 3).

Variables	Items	Number	Percentage
Sex	Male	115	40%
	Female	173	60%
Age	15–19 years old	14	5%
	20–24 years old	89	31%
	25–29 years old	123	43%
	30–34 years old	62	21%
Occupation	University student	87	30%
	Company employee	120	42%
	College teacher	35	12%
	others	46	16%
Education level	Undergraduate	179	62%
	Graduate student or more	109	38%

#### Results and discussion

##### Manipulation check

There was a significant difference between the three groups (M_once_ = 1.90, M_three-times_ = 3.70, M_seven-times_ = 6.30, *t* = 22.76, *p* < 0.001). Our manipulation of the marketing intensity was successful.

##### Purchase intention

Then, marketing intensity was set as three dummy variables (0 = once a week, 1 = three times a week, 2 = seven times a week) to conduct a logistic regression analysis. First, ANOVA on purchase intention showed a significant effect of marketing intensity [*F*(1,287) = 80.63, *p* < 0.001]. Second, ANOVA on cognitive fluency showed a significant effect of marketing intensity [*F*(1,287) = 17.22, *p* < 0.001]. Third, cognitive fluency positively affected customer inspiration [*F*(1,287) = 317.76, *p* < 0.001] and negatively affected perceived risk [*F*(1,287) = 213.24, *p* < 0.001]. Fourth, there was a significant effect of customer inspiration, perceived risk, and cognitive fluency as dependent variables on purchase intention [*F*(3,285) = 374.83, *p* < 0.001].

**Figure 2:**
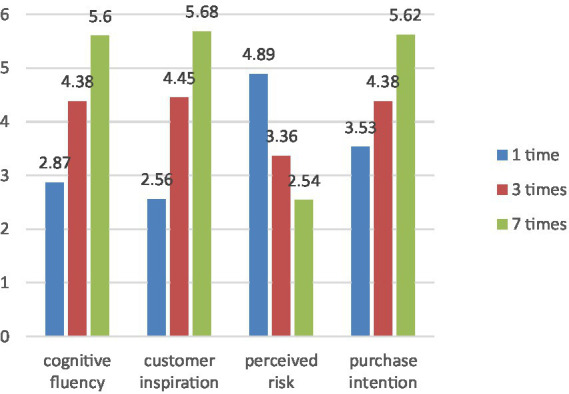
Mediation effect of customer inspiration and perceived risk.

Specifically, when we marketed plant-based meat seven times a week, this group had the highest cognitive fluency (M_once_ = 2.87, M_three-times_ = 4.38, M_seven-times_ = 5.60, *p* < 0.001), customer inspiration (M_once_ = 2.56, M_three-times_ = 4.45, M_seven-times_ = 5.68, *p* < 0.001), and purchase intention (M_once_ = 3.53, M_three-times_ = 4.38, M_seven-times_ = 5.62, *p* < 0.001) than others. The group which marketed plant-based meat once a week had the highest perceived risk (M_once_ = 4.89, M_three-times_ = 3.36, M_seven-times_ = 2.54, *p* < 0.001; Figure 2).

##### Mediation effect

The indirect effect of social media marketing intensity on purchase intention was significant via cognitive fluency, with 95% confidence intervals excluding 0 (Model 4; β = 0.83, Se = 0.09; 95% CI: 0.66–1.01), showing further support for H2. In addition, the indirect effect of “social media marketing intensity →cognitive fluency →customer inspiration→ purchase intention” (β = 0.10, Se = 0.08; 95% CI: 0.00–0.14) and “social media marketing intensity →cognitive fluency →perceived risk→ purchase intention” were significant (β = 0.13, Se = 0.12; 95% CI: 0.04–0.27). The direct effect of social media marketing intensity on purchase intention was significant (β = 0.22, Se = 0.10; 95% CI: 0.02–0.42). That is to say, customer inspiration and perceived risk play a mediating role between social media marketing and purchase intention. In addition, a higher level of cognitive fluency is more likely to generate customer inspiration and improve purchase intention. In comparison, a lower level of cognitive fluency is more likely to stimulate perceived risk, thus reducing purchase intention. Therefore, H3 and H4 have been supported.

### Study 4: Brand community identity as a boundary condition

#### Design and method

To deepen our framework further, we designed Study 4 to include brand community identity as a boundary condition (H5 and H6), which was well recognized as an individual status feature. We chose Beyond Meat from study 1 as a stimulus and conducted this experiment online.

Study 4 adopted a 2 (brand community identity: in-group vs. out-group) × 3 (marketing intensity: once a week vs. three times a week vs. seven times a week) between-subjects design with brand community identity as an additional measured factor. First, we used the “Beyond Meat” forum on Microblog as a brand community. In-group members were fans from “Beyond Meat” forum, while recruited out-group members from the Questionnaire site. We also included the questions such as “To what extent do you think you belong to this brand” (1 = fully in; 7 = not at all) to test the validity of the members’ identity. We sent invitations to the members, and a total of 300 participants (182 females, M_age_ = 27.65, ranging from 17 to 36 years) were willing to participate in the study for a small payment. There were 150 participants in the in-group and 150 participants in the out-group.

Next, we manipulated social media marketing intensity according to Study 3. We set up three WeChat groups, and the in-group and out-group participants were randomly assigned to three WeChat groups, with 100 participants each. We sent Beyond Meat’s product information to three groups weekly, three times a week, and seven times a week, respectively. In particular, the conditions were the same except for the frequency at which sent the manipulated material ads.

After a week, participants were asked to fill in the questionnaire for “marketing intensity,” “cognitive fluency” (α = 0.95), “customer inspiration” (α = 0.92), “perceived risk” (α = 0.92), and “purchase intention” (α = 0.91) similar to prior studies. The questionnaire also included the question “To what extent do you consider yourself a member of this brand community” to confirm members’ identity. Meanwhile, study 4 required participants to provide demographic information. There were 292 valid questionnaires in study 4 (178 females, M_age_ = 27.55, SD = 3.31).

#### Results and discussion

##### Manipulation check

T-test results showed significant differences between in-group and out-group (M_in-group_ = 6.00, M_out-group_ = 1.51, *t* = 49.79, *p* < 0.001), indicating successful variable manipulation. In-group members considered themselves as members of the “Beyond Meat” forum, while out-group members considered themselves not fans of the “Beyond Meat” forum. The manipulation of marketing intensity was successful (M_once_ = 1.82, M_three-times_ = 3.45, M_seven-times_ = 6.70, *p* < 0.001).

##### Purchase intention

First, ANOVA on purchase intention revealed a significant main effect of cognitive fluency [*F*(1,298) = 15.61, *p* < 0.001]. Second, the interaction between cognitive fluency and in-group positively affected customer inspiration [*F*(1,298) = 12.68, *p* < 0.001]. Meanwhile, the interaction between cognitive fluency and in-group significantly influenced purchase intention [*F*(1,298) = 6.67, *p* < 0.001]. Third, the interaction effect between cognitive fluency and out-group on perceived risk was significant [*F*(1,298) = 7.34, *p* < 0.01]. In addition, the interaction between cognitive fluency and out-group significantly influenced purchase intention [*F*(1,298) = 14.26, *p* < 0.001].

**Figure 3:**
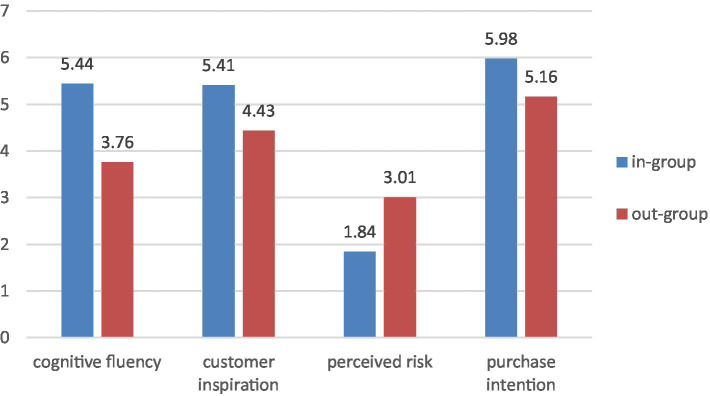
Moderating effect of brand community identity.

Specifically, in-group members had higher cognitive fluency (M_in-group_ = 5.44, M_out-group_ = 3.76, *t* = 13.85, *p* < 0.001) and purchase intention (M_in-group_ = 5.98, M_out-group_ = 5.16, *t* = 7.93, *p* < 0.001) than out-group members. In addition, in-group members had higher customer inspiration (M_in-group_ = 5.41, M_out-group_ = 4.43, *t* = 6.85, *p* < 0.001), while out-group members had higher perceived risk (M_out-group_ = 3.01, M_in-group_ = 1.84, *t* = 8.92, *p* < 0.001; Figure 3).

##### Mediation effect

We used Model 7 (Hayes, 2013) to examine the moderated mediation effect in study 4. The results showed that the interaction effect of cognitive fluency and in-group on purchase intention was significant (β = 0.20, *p* < 0.05, 95% CI: 0.06–0.86), and the interaction effect of cognitive fluency and out-group on purchase intention was also significant (β = −0.68, *p* < 0.05, 95% CI: −1.16 to −0.12). The above results verified the existence of the moderated mediation effect in this study. In-group members are more likely to generate higher cognitive fluency and customer inspiration, further enhancing purchase intention. Out-group members are more likely to generate lower cognitive fluency and perceived risk, reducing purchase intention. Thus, H5 and H6 were supported.

## General conclusion and discussion

### Research conclusion

This research examines how and why social media marketing of plant-based meat can affect young people’s purchase intention. The results indicate that social media marketing generally obtains higher purchase intention, increasing marketing intensity in the plant-based meat promotion phase. Cognitive fluency is the important mediating variable linking social media marketing with consumers’ purchase intention. This is because plant-based meat as a new product is unfamiliar to most Chinese, and the more frequent interaction on social media leads to increased cognitive fluency, attracting young people’s intention.

We also verified that two motivations drove young people’s purchase intentions: customer inspiration and perceived risk. After obtaining plant-based meat information, young people’s purchase intention depends on which customer inspiration or perceived risk is dominant. When cognitive fluency is high, individuals are more likely to generate customer inspiration and improve their purchase intention. Conversely, a lower level of cognitive fluency is more likely to stimulate perceived risk and reduce young people’s purchase intention.

Furthermore, brand community identity plays a moderating role in consumers’ perception and purchase processing. Members’ status will affect whether they perceive plant-based meat marketing as more imaginative or risky. In-group members have stronger cognitive fluency in plant-based meat than those out-group members. In addition, in-group members are more likely to generate customer inspiration and enhance purchase intention, while out-group members tend to stimulate perceived risk and reduce purchase intention.

### Theoretical contributions

Our study makes theoretical contributions to the existing research in several ways. First, this paper contributes to the literature on cognitive fluency, explaining why social media marketing of plant-based meat can increase young people’s purchase intention more than traditional marketing. Based on dual-system theory, we clarify the cognitive evaluation mechanisms between plant-based meat marketing and purchase intention such that individuals’ cognitive fluency will influence their behavioral decisions. Although previous studies have demonstrated the factors influencing the marketing of plant-based meat, the micro research on young people’s cognition in the context of social media has been neglected (Loo et al., 2020; Zhao et al., 2022). Our finding directly responds to the call that “the basic process of plant-based meat evaluation and special marketing context has still been ignored” (Qutteinq et al., 2019; Bryant and Sanctorum, 2021) by explaining how young people make decisions to adopt plant-based meat.

Second, by proving that the subsequent influence of cognitive fluency on purchase intention depends on customer inspiration or perceived risk, which is the dominant factor, we clarify the psychological mechanism of young people processing plant-based meat. Previous studies on plant-based meat have concluded that consumers keep a single positive or negative attitude (McCarthy et al., 2015; Bryant et al., 2018). However, this paper proves that consumers have two opposite cognitions of plant-based meat simultaneously, providing a new theoretical basis for studying plant-based meat purchases. In addition, this paper integrates the concepts of customer inspiration and perceived risk into the research framework of the cognitive evaluation process in the context of social media marketing for the first time, further enriching the dual-system processing theory.

Moreover, our study focuses on the role of consumers’ states in the perception and decision-making process, revealing the moderating role of brand community identity. Prior research has found that brand identity leads to more fabulous inspiration (Algesheimer et al., 2005) and a more positive mood (Chadborn and Reysen, 2018). We contribute to this line of research by showing that in-group members with brand community identity have higher customer inspiration and purchase intention. In contrast, out-group members can enhance perceived risk and reduce purchase intention in the social media marketing. This response to the statement that “there should be a greater understanding of the reasons that hinder consumer acceptance of plant-based meat” (He et al., 2020). More importantly, the different perceptions and decisions of in-group members and out-group members are also consistent with dual-system theory, thus contributing to dual-system theory.

### Practical implications

There are also some implications for entrepreneurship practice. First, consumers generally avoid sloppy information and prefer smooth product introduction (Hu et al., 2017; Graf et al., 2018). Thus, managers can look for ways to reduce young people’s perception of effort and improve their fluency experience when designing plant-based meat advertisements, using our findings in which social media marketing improves young people’s preferences more than traditional marketing. For example, with the help of artificial intelligence and system algorithms, social media marketing can choose different forms of information presentation, such as pictures and videos, according to young people’s cognitive habits. In addition, managers can increase user interaction as much as possible to meet young people’s social behaviors through plant-based meat’s healthy and environmental protection products.

Second, young people have different perceptions of inspiration or risk for plant-based meat. On the one hand, customer inspiration can significantly improve purchase intention for plant-based meat. Therefore, enterprises adopt the traditional marketing model that mainly satisfies the individuals’ basic requirements and introduce the marketing model that is inspired by young people’s potential needs. Enterprises can design the packaging and formulation of plant-based products to be more open. In addition to the traditional evaluation of plant-based meat, such as appearance, practicality, and willingness to pay, can add the index of consumers’ inspiration. On the other hand, managers also need to pay attention to negative effects and reduce risk perception by increasing information fluency. Companies thus can provide targeted information to different innovative individuals.

Third, to provide substantial value for managers and offer them actionable implications, we also tested the impact of community membership status. Community members are mostly existing, or potential brand users, and they are the preferred channels for enterprises to request participation and feedback on plant-based meat ideas. Considering that the community can significantly reduce customers’ risk perception, improving the positive impact of cognitive fluency on customers’ inspiration. When launching new plant-based products, enterprises can preferentially select in-group customers to try them out, capturing insights and opinions through user-generated content analysis. Meanwhile, managers can also carry out brand community publicity, attracting out-group members to join through vouchers and discounts.

### Limitations and future research

Some limitations of this study suggest avenues for further research. First, plant-based meat as a new product is updated quickly, and the experimental materials cannot cover all categories of plant-based products. Social media marketing of different types of plant-based products may have other effects. For example, the fluency experience of hedonic products can increase product purchase, while it does not influence practical products (Shen et al., 2016). In other words, consumers have different perceptions of plant-based meat’s functions, leading to opposite purchase intentions. Therefore, it is necessary to consider whether plant-based meat is marketed for environmental or practical purposes. We welcome further research to address this exciting research question with different product types.

The research samples and method in this paper may have some limitations. As this paper mainly adopts lab study, the sample size is relatively small. It would be difficult to predict all young Chinese’ attitude toward plant-based meat. Follow-up studies are expected to use larger sample sizes. In addition, in manipulating the traditional marketing of plant-based meat and social media marketing, this paper has made efforts to control the consumers’ characteristics that may affect their purchase intention. However, “participants can post comments after the post” in the social media marketing condition will lead to participants’ engagement and further change their purchase intention. These interfering factors may affect the validity of the conclusion. Therefore, improving research methods and enhancing research validity are essential for future efforts.

Although we have built a basic model about the social media marketing of plant-based meat on purchase intention and explored the path of mediation, we encourage researchers to study the mediation mechanism through more scientific methods. This paper finds that customer inspiration and perceived risk are parallel intermediary processes, which can produce opposite results on purchase intention. But the extent of their effects is still unclear. For instance, it is possible that purchase intention actually causes increases in inspiration through a dissonance mechanism. For the research model with multiple mediator variables, it is hoped that future scholars can conduct strong inferences about the order of the effects.

Lastly, our research about social media marketing of plant-based meat on purchase intention is based on a general situation. Individuals need to use visual, tactile, auditory, and other senses to obtain product information when evaluating products (Troye and Supphellen, 2013). Different advertising designs have other sensory stimuli for consumers. It is hoped that more dimensions of social media marketing and advertising designs (text, pictures, animation, exhibition, et al.) can be tapped as independent variables for further exploration. In addition, does over-marketing of plant-based meat have the opposite effect on younger? How do shopping tasks with different degrees of difficulty affect young people? Under what circumstances can young people produce a negative shopping mood? These questions offer directions for further research among both academics and practitioners.

## Data availability statement

The raw data supporting the conclusions of this article will be made available by the authors, without undue reservation.

## Ethics statement

Ethical review and approval was not required for the study on human participants in accordance with the local legislation and institutional requirements. Written informed consent from the patients/participants or patients/participants legal guardian/next of kin was not required to participate in this study in accordance with the national legislation and the institutional requirements.

## Author contributions

All authors listed have made a substantial, direct, and intellectual contribution to the work and approved it for publication.

## Funding

This research was supported by the National Social Science Foundation of China (21BGL131).

## Conflict of interest

The authors declare that the research was conducted in the absence of any commercial or financial relationships that could be construed as a potential conflict of interest.

## Publisher’s note

All claims expressed in this article are solely those of the authors and do not necessarily represent those of their affiliated organizations, or those of the publisher, the editors and the reviewers. Any product that may be evaluated in this article, or claim that may be made by its manufacturer, is not guaranteed or endorsed by the publisher.
